# Unveiling Synergistic Interface Effects on Charge Trapping Regulation in Polymer Composite Dielectrics through Multiscale Modeling

**DOI:** 10.1021/acs.jpcb.4c08661

**Published:** 2025-04-23

**Authors:** Haoxiang Zhao, Lixuan An, Daning Zhang, Xiong Yang, Huanmin Yao, Guanjun Zhang, Haibao Mu, Björn Baumeier

**Affiliations:** † State Key Laboratory of Electrical Insulation and Power Equipment, School of Electrical Engineering, Xi’an Jiaotong University, Xi’an 710049, China; ‡ Department of Mathematics and Computer Science, Eindhoven University of Technology, P.O. Box 513, 5600MB Eindhoven, The Netherlands; § Institute for Complex Molecular Systems, Eindhoven University of Technology, P.O. Box 513, 5600MB Eindhoven, The Netherlands; ∥ KERMIT, Department of Data Analysis and Mathematical Modelling, Ghent University, 9000 Ghent, Belgium

## Abstract

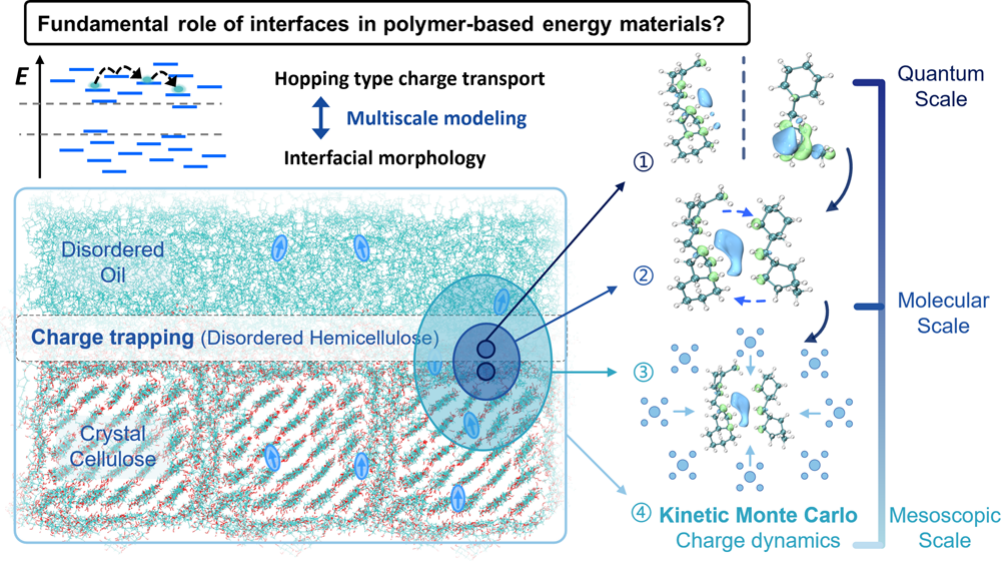

Interface design is a promising strategy to enhance the dielectric strength in polymer composites through regulating the charge transport process. However, the targeted exploitation of interface effects is limited due to a lack of fundamental understanding of the underlying mechanisms involving elementary electronic processes and details of the intricate interplay of characteristics of molecular building blocks and the interfacial morphology – details that cannot fully be resolved with experimental methods or commonly used band transport models. Here, we instead build a proper theoretical framework for polymer dielectrics based on charge hopping and employ a multiscale modeling approach linking the quantum properties of the charge carriers with nano- and mesoscale structural details of complex interfaces. Applied to a prototypical application-proven cellulose-oil interface system, this approach demonstrates that charges are trapped in the disordered region. Specifically, it unveils this trapping as a synergistic effect of two transport-regulating interface mechanisms: back-transfer to the oil region is suppressed by energetic factors, while forward-transfer to the crystalline cellulose is suppressed by low electronic coupling. The insight into the molecular origins of interface effects via dual-interface regulation in the framework of charge hopping offers new development paths for developing advanced energy materials with tailored electrical properties.

## Introduction

Interfaces play a pivotal role in enhancing the performance of materials, particularly in improving the dielectric strength of polymer dielectrics used in capacitive energy storage and electrical engineering applications.^1−7^ Since T. J. Lewis’s milestone introduction of the “nanodielectrics” concept in 1994, interface effects have been recognized as crucial in improving dielectric strength,^8−11^ and interface design has been regarded as an effective practical strategy.^12−15^ Although it is widely acknowledged that the dielectric strength is primarily determined by charge transport processes,^8−11,16−18^ many of its improvements are attributed to the hindrance of charge transport,^18−37^ the field lacks a fundamental understanding of interface effects on charge transport, its underlying mechanisms, and even the basic microscopic dynamic properties. Consequently, practical design rules for guiding material design are speculative at best.

Nevertheless, progress has been made in addressing practical issues in the development and engineering of interfaces in polymer-based energy storage dielectrics design.^12−15^ This includes various nanodoping techniques^12,19−23^ and hierarchical interface designs.^12,24−28^ However, these applications largely remain trial-and-error,^13,32^ and a unified strategy has yet to emerge. Stronger yet, inconsistent or contradictory explanations of the interface effect mechanism are also reported.^13^ The choice of a suitable model for the charge transport mechanism is essential to resolve these issues. Mainstream interfacial theoretical models based on the electrical double-layer model assume that accumulated charge inhibits subsequent charge transport,^8−10,38^ which is premised on the strong conduction known for ordered solids. Similarly, other discussions of the charge transport mechanism focus on the bandgap and trap levels inside it. However, it is debatable if the underlying band-type charge transport framework, which is suitable for crystals, is appropriate for polymer composite dielectrics. In crystalline systems, the highly ordered structures enable the formation of energy bands, allowing charges to move (nearly) freely through spatially extended bands after excitation across the bandgap. In contrast, the structural disorder of polymer dielectrics leads to localized electronic states, typically necessitating charge transport via hopping.^39−41^ Particularly, Alan J. Heeger presented in his Nobel lecture the understanding of the hopping-type charge transport in polymeric and organic materials, referring to the Marcus theory,^42,43^ which is expected to be a promising theoretical framework for the charge transport in polymer dielectrics.

Fundamentally, challenges in understanding interface effects on charge transport stem from the limitations in resolving the dynamic charge transport process from the nano- to macroscale (or at least mesoscale) and thereby in shedding light on the interplay between the chemistry of the molecular building blocks and the local and global structural features (morphology) of the interfaces. On the one hand, state-of-the-art experimental techniques, like pulsed electric-acoustic current or thermal simulated depolarization current, can only reflect the whole sample’s overall charge distribution or trap characteristics without microscopic dynamic details.^13,18,36,37,44,45^ Based on these macroscopically measured bulk properties of the material, it is difficult to even distinguish between the contributions from the newly added component itself or the created interface. On the other hand, quantum chemistry (QC) calculations, e.g., based on Density Functional Theory (DFT), offer nanoscale electronic structure insights^6,13,34,36−38,44,45^ but fail to capture charge dynamics in a complex interface structure at a larger scale. The limited understanding of charge transport characteristics results in interface effects being treated as a black box problem, leading to speculative or hypothetical theoretical models at best. A bridge connecting dynamic charge transport and structural material properties across scales is urgently needed.^13,46^

Multiscale modeling offers such a bridge to provide unprecedented insight into the interface effects on charge transport.^47−49^ Combining molecular dynamics (MD) simulations to obtain atomic-level resolution of an interface morphology and large-scale embedded quantum chemistry calculations, our bottom-up multiscale approach allows for a predictive evaluation of the electronic properties of the individual molecular building blocks and their interactions as they enter the Marcus rate for electron-transfer theory. With this, parameter-free charge transport simulations are realized within a realistic morphology as rate-based dynamics using kinetic Monte Carlo (KMC) methods. Such a combined approach covers the interplay of the two key aspects – realistic interface structure and dynamic charge transport, promising an understanding of interface effects from nano to macro/mesoscale.

To demonstrate the advantages of this approach, we are using the classic cellulose-oil composite as a study subject. Its dielectric strength is significantly increased by the cellulose-oil interface introduced through the simple impregnation of porous cellulose dielectric paper with oil.^31,50,51^ Widely used in electrical engineering, its reliability stands the test of several decades during the electrification process.^7,52^ Through multiscale modeling, this work aims to grasp the characteristics of interface-related charge transport, explore the mechanisms of interface effects on charge transport regulation, and offer practical guidance for dielectric interface design. To this end, first, a representative cellulose-oil interfacial morphology is modeled on atomic resolution, containing two typical interface types in dielectrics design: one between oil and disordered hemicellulose and one between hemicellulose and crystalline cellulose. Then, a combination of quantum and classical methods (see “Methods” section) is used to evaluate the physical quantities entering the Marcus hopping rate, i.e., the ionization energy of each molecular unit (measuring its charge-attracting ability), also known as site energy, and the strength of the electronic coupling between neighboring units, as they are influenced by structural disorder. Finally, the charge dynamics are obtained on the whole oil-cellulose system and further analyzed in terms of the influence of site energy differences, external driving force, and electronic coupling for both interface types.

Results unveil remarkable trap characteristics of the cellulose-oil interface: once the charge enters the region between two interfaces, it hardly escapes by either back-transfer recrossing the interface 1 or forward-transfer crossing the interface 2 to the crystalline cellulose. The trapping of charges in the hemicellulose region is, therefore, efficiently regulated by the synergy of both interfaces rather than by a single interface. Detailed analysis also reveals different regulatory mechanisms at the two interfaces, where the trapping at the oil-hemicellulose interface is regulated by differences in site energies, whereas the trapping at the hemicellulose-cellulose interface is regulated by low electronic coupling across it. While energy-regulated trapping is a widely recognized concept, its synergy with the crucial role of coupling-regulated trapping in the emergence of what is referred to as the interface effect has previously been overlooked.

Unveiling and then utilizing the synergy between these two regulating mechanisms and their molecular origin allows for a more flexible approach to interface engineering. The insights gained from our findings not only broaden the view on the origins of the interface effects, thereby offering more reasonable explanations for various successful advanced material modifications,^32−37^ but may unify related dielectric material innovation at a higher level.

## Methods

### Interfacial Morphology Construction by Molecular Dynamics

**Figure 1:**
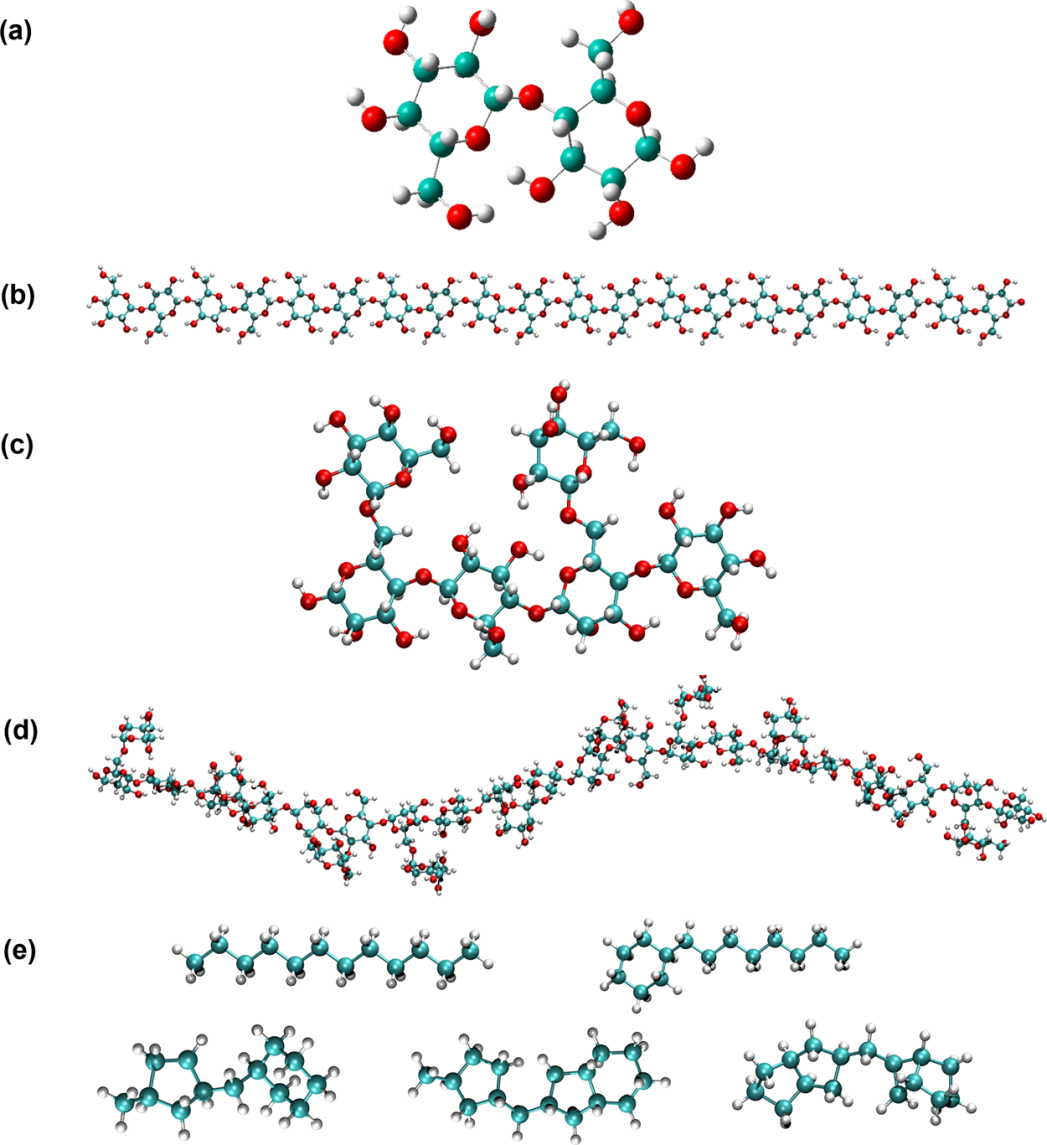
Chemical structures of cellulose and oil. (a) Cellobiose. (b) Cellulose molecule. (c) Repeating unit of hemicellulose. (d) Hemicellulose molecule. (e) Oil molecules.

The chemical structures of all molecules are shown in Figure 1, referring to previously published studies.^53−60^ Cellobiose, a reducing sugar, is a disaccharide with the formula C12H22O11 and consists of two d-glucose molecules linked by a β(1 → 4) bond as shown in Figure 1a. The cellulose molecular chains are made up of 20 linked d-glucose units in this way, as shown in Figure 1b. The detailed structure of the repeating unit of hemicellulose is shown in Figure 1c. This branched polysaccharide is composed of d-glucose, D-galactose, and D-mannose residues. As the linking core, the mannose is β(1 → 4) linked to a d-glucopyranose residue, while simultaneously forming an α(1 → 6) glycosidic bond with a D-galactopyranose unit, creating a branch at the C6 position of the mannose. In this way, hemicellulose is formed by 10 repeating trisaccharide units as shown in Figure 1d. The choice of the molecule length follows the previous related work,^53,55,58−60^ where the molecule lengths from 10 to 40 are found independent of structure conformation and physicochemical properties such as density, order degree, mechanical stress, etc.^53,55,58−60^ In order to reflect the long chain structure of cellulose and to reduce the amount of calculation for subsequent quantum chemical calculations, we selected the middle length of 20. The chemical structures (shown in Figure 1e) and quantitative ratio of the oil components and the subsequent relaxation are based on refs (57,58). There are 5 different oil molecule types, which are with two types of molecular groups: cycloalkanes and chain alkanes.

The cellulose-oil interfacial model consists of oil and repeating microfibrils. The molecular dynamics (MD) is carried out with the GROMACS package^61^ and the OPLS-AA force field,^62^ velocity-rescale thermostat (time constant 0.2 ps), and Berendsen barostat (time constant 0.5 ps). The MD construction process of microfibril is shown in Figure 3b. First, the crystal cellulose nanofibril core of the microfibrils is constructed by the Cellulose Builder package.^63^ Small-angle neutron scattering and wide-angle X-ray scattering studies^64−66^indicate that, depending on the nature of cellulose, its microfibrils can contain between 12 and 36 chains. Here we model a crystalline core consisting of 25 cellulose chains (arranged in a 5 × 5 configuration), which is well compatible with the experimentally available data. Next, the crystal core is combined with the surrounding hemicellulose to form a microfibril. The detailed MD relaxation process of the microfibril refers to the refs (53−56). First, the heterogeneous structure is energy-minimized. This is followed by a constant volume and temperature (*NVT*) ensemble for 5 ns with the thermostat set to 450 K. Next, without changing the thermostat, the barostat is set to 10 bar (*NPT*), and the atoms are simulated for 10 ns. Finally, the structure is relaxed at 300 K under 1 bar for another 10 ns. The time step is 0.002 ps. An increased temperature typically accelerates the simulation and, in this case, helps disordered hemicellulose to find its equilibrium position. However, to not disorder the crystalline cellulose, its atoms are position-restrained when the system is at high temperature. Then, the oil molecules are combined with the repeating microfibrils, and the disordered oil region is relaxed, referring to previous studies^57,58^ while the other part is restrained. Based on the amorphous loose structure characteristics of hemicellulose and the engineering practice of full impregnation, the compatibility of oil and hemicellulose is high. The phase separation between oil and disordered hemicellulose is not very strong, and the interface could be defined based on different regions of molecules rather than the interfacial shape.

The relaxation process involves 5 ns under the *NVT* ensemble at 450 K, 10 ns under the *NPT* ensemble at 450 K and 10 bar, and 5 ns under the *NPT* ensemble of 300 K and 1 bar. Finally, the whole interfacial system is relaxed at 300 K under 1 bar for 10 ns.

### Quantum Chemistry Calculations

For all the molecules/segments used in this work, DFT calculations (geometry optimization and single-point calculations) are performed with the hybrid PBE0^67^ functional with D3BJ dispersion correction and the def2-tzvp^68^ basis set using the ORCA quantum chemical package.^69^ Additionally, the analysis and visualization of results are based on the software Multiwfn.^70^

### Marcus Rate Calculation and Multiscale Integration

After the interfacial morphology is constructed, it is then partitioned into hopping sites, and the Marcus charge hopping rates between site pairs are calculated separately based on eq 8, where multiscale factors are integrated. The definition of site pairs is based on a fragment closest contact threshold (0.65 nm), ensuring that all possible hopping processes are systematically incorporated into our charge transport analysis. Then, the charge transport dynamics can be realized by the kinetic Monte Carlo method. This stepped multiscale process is mainly carried out based on the open-source software VOTCA-XTP.^48,71^ This software provides interfaces for mainstream molecular dynamics and quantum chemistry calculation packages, realizing a complete solution for charge transport simulation in complex molecular systems.

Based on the Marcus theory,^72^ the charge hopping rate ω_*ij*_ from site *i* to *j* can be calculated according to eq 8, where *J*_*ij*_ is the electronic coupling between the initial and final electronic states, λ_*ij*_ is the reorganization energy, Δ*E*_*ij*_ is the site-energy difference, *ℏ* is the reduced Planck constant, *k*_B_ is the Boltzmann constant and *T* is the temperature. The Marcus rate captures and combines multiscale influence factors via *J*_*ij*_, λ_*ij*_, and Δ*E*_*ij*_, which are all obtained from first-principles calculations in this study.

The site energy difference, Δ*E*_*ij*_ = *E*_*i*_ – *E*_*j*_, drives the charge transfer between site *i* and *j*. Multiscale factors are introduced and combined in this work through summation of all contributions due to internal energy differences, externally applied electric field, electrostatic interactions, and polarization effects:
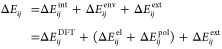
1Note that in the “Results and Discussion” section below, we identify the site energy difference with the difference of ionization potentials in the case of hole transport. In the case of electron transport, this would correspond to the difference in the negative electron affinities.

**Figure 2:**
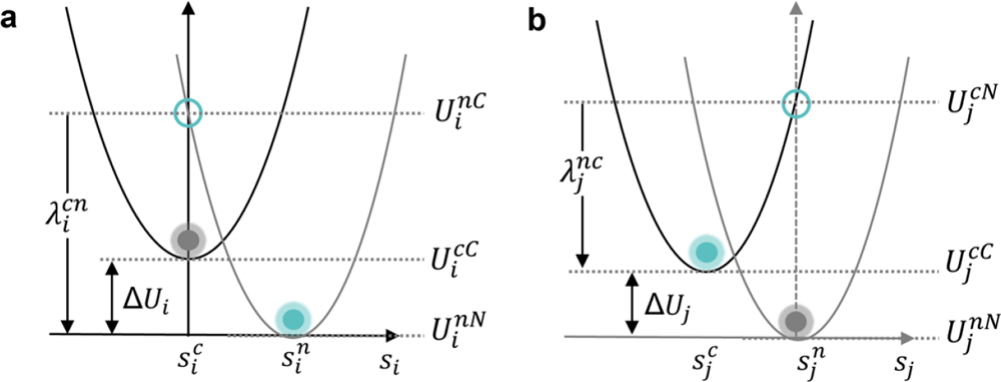
Schematic diagram of energies used for charge transport rate calculation through a four-point method. (a) Donor and (b) Acceptor in charged and neutral states. *U*_*i*_^nC^ is the total energy of site (segment) *i* in the neutral state and charged geometry (small *n* denotes the state and capital *C* the geometry).

The internal energy difference Δ*E*_*ij*_^int^ is the contribution at the quantum scale and can be calculated using DFT according to
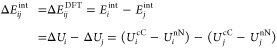
2where *U*_*i*_^cC(nN)^ is the total energy of site (segment) *i* in the charged (neutral) state and geometry obtained from DFT. In a similar fashion, the reorganization energy is calculated based on Nelsen’s four-point method^73^ shown in Figure 2:
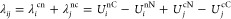
3

The effects of the environment on the energy differences, Δ*E*_*ij*_^env^, are in our microelectrostatics approach, composed of electrostatic (Δ*E*_*ij*_^el^) and polarization (Δ*E*_*ij*_^pol^) contributions. The electrostatic contribution to the energy of site *i* is determined from atomic partial charges as
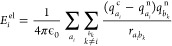
4where *r*_*a*_*i*_*b*_*k*__ is the distance between atoms *a*_*i*_ and *b*_*k*_, and the *q*_*a*_*i*__^n(c)^ are the partial charges of atom *a* in segment *i* in state n or c. Polarization effects are incorporated at atomistic resolution. Specifically, here we further use a polarizable force field with atomic dipole polarizabilities. The induced dipoles **μ**_*a*_*i*__ are obtained iteratively by
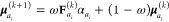
5where **F**_*a*_*i*__^(*k*)^ is the evaluated electric field at atom *a* in molecule/segment *i* by all atomic partial charges and induced moments, and α_*a*_*i*__ is the isotropic atomic polarizability. The parameter ω is a damping constant for successive over-relaxation. All details about the related calculations can be found in a previous study.^48^ Long-range electrostatic interactions are accounted for via a periodic embedding of aperiodic excitations based on Ewald summation.^49^ Polarization effects are considered within a cutoff of 3.0 nm around each individual segment.

Finally, Δ*E*_*ij*_^ext^ is the contribution due to the site-energy difference from the external electric field **F**^ext^:
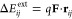
6where *q* is the charge and **r**_*ij*_ is the vector connecting sites *i* and *j*.

The electronic coupling *J*_*ij*_ expresses the coupling strength between two electronic states localized on sites *i* and *j*, respectively, and is defined as
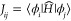
7where ϕ_*i*_ and ϕ_*j*_ are the molecular orbital wave functions of the related electronic states, respectively, and *Ĥ* is the Hamiltonian of the dimer. Within the frozen-core approximation, the usual choice for the diabatic wave functions is the frontier orbitals. Equation 7 is evaluated in this work using the dimer projection method.^47^

## Results and Discussion

### Cellulose-Oil Interface and Multiscale Model

**Figure 3:**
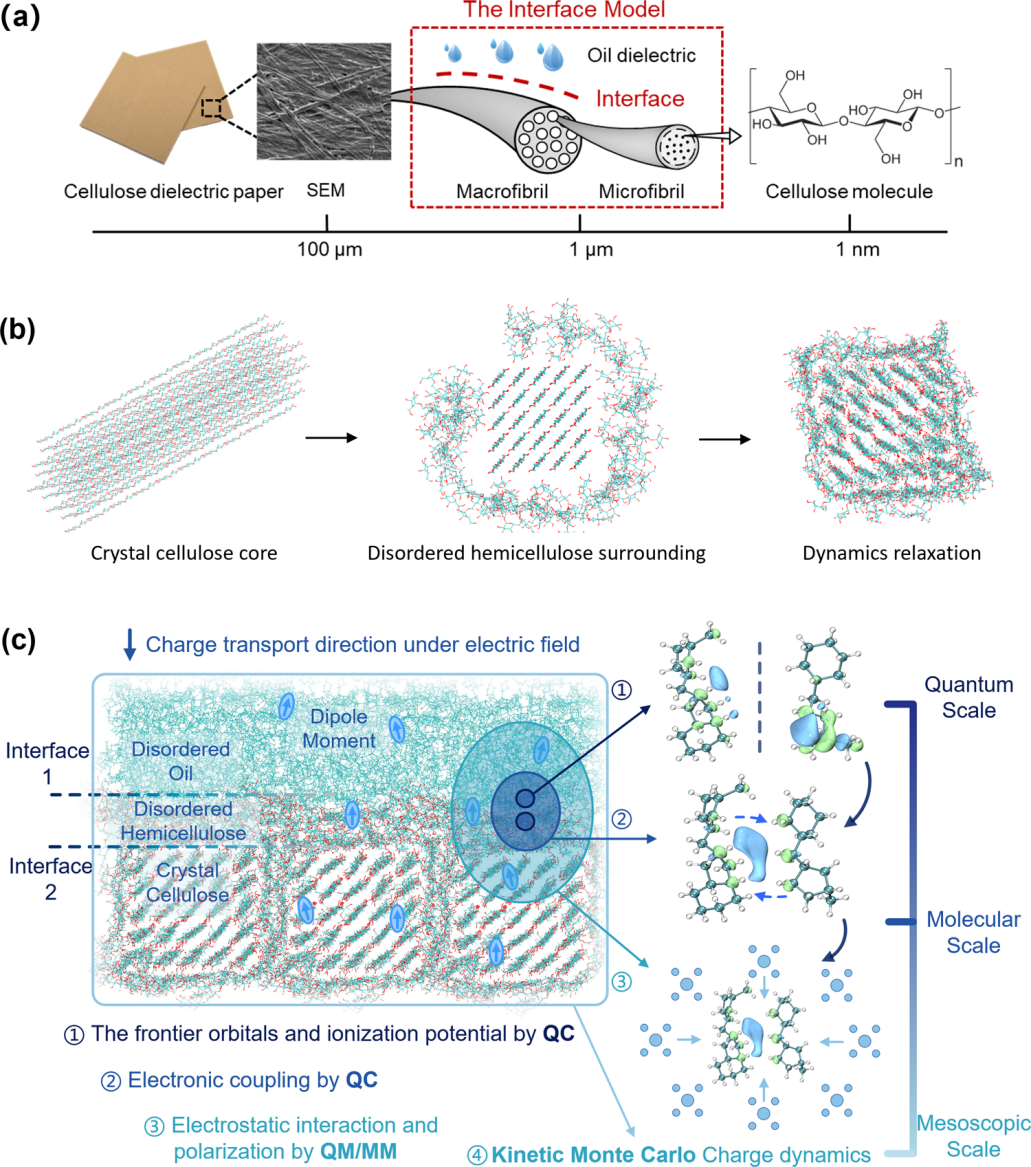
Cellulose-oil interface multiscale modeling. (a) Conceptualization of the cellulose-oil interfacial model. (b) MD model of cellulose microfibril, with a crystalline cellulose core surrounded by disordered hemicellulose. (c) Schematic of the elements of the multiscale method in the constructed interfacial morphology.

The first step in the multiscale model of charge dynamics for localized-state polymer dielectrics is the simulation of a realistic, representative morphology of a cellulose-oil interface with MD. The structural model we adopt here is based on multiscale deconstruction analysis,^53−55^ as shown in Figure 3a: From the cellulose dielectric paper, macrofibrils can be observed at the 100 μm scale. Each macrofibril is composed of repeating microfibrils of 1 μm scale. Ultimately, microfibrils consist of cellulose molecular chains at the nanoscale. In detail, the core of the microfibril is crystalline cellulose, surrounded by disordered hemicellulose (Figure 3b). The final constructed cellulose-oil interfacial model includes oil and repeating microfibrils (left part in Figure 3c), and computational details on MD modeling are provided in the “Methods” section. There are two typical interfaces along the electric field. Interface 1 is between oil and disordered hemicellulose, which can represent an interface formed by two different materials, and the mechanism subsequently revealed can apply to the nanodoping interface. Interface 2 is between disordered hemicellulose and crystalline cellulose, which can represent an interface formed by two similar materials, and the mechanism can apply to the interface in all-organic composites.

After the interfacial morphology model is simulated, it is partitioned into hopping sites (segments/monomers) for charge transport simulation. The charge dynamics model starts from a single charge hopping (or electron transfer event) between a pair of sites, integrating factors of different scales, and builds charge transport in the interfacial morphology as a sequence of such bimolecular transfers. The logic flow is as shown from ① to ④ in the right part of Figure 3c. Based on the Marcus theory for hopping-type charge transfer in localized-state dielectrics,^39−43^ the bimolecular hopping rate ω_*ij*_ between two sites *i* and *j* can be calculated according to
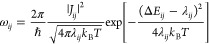
8and depends on two crucial factors: the site energy difference Δ*E*_*ij*_ and the electronic coupling *J*_*ij*_ (other parameters in eq 8 are introduced in the “Methods” section). The former is determined by the individual charge-attracting abilities (ionization potentials) or site energies of the monomers *E*_*i*_, which comprise internal quantum-mechanical (from the chemistry of the material) and external electrostatic contributions (from the morphology), as well as contributions from an external electric field. The electronic coupling *J*_*ij*_ ‘bridges’ two monomers and is a measure of the quantum-mechanical interaction between site-localized electronic states. Both quantities will be analyzed below. Based on the Marcus theory, not only the multiscale factors can be integrated during the calculation of hopping rates, but also the charge transport in localized-state dielectrics is discussed in a complete and appropriate theoretical framework.

### Charge Trapping and Its Regulation by a Dual-Interface Effect

**Figure 4:**
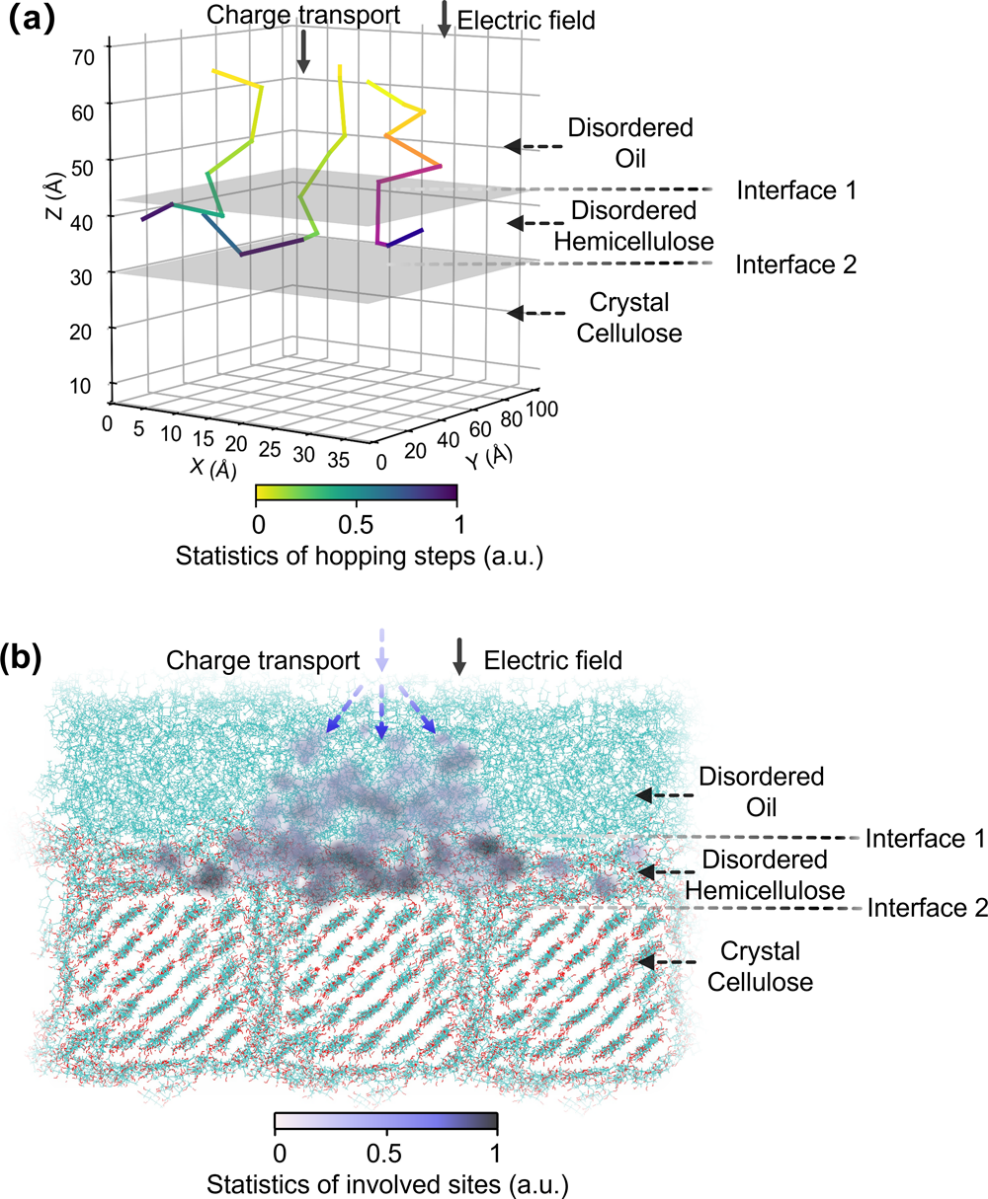
Intuitive charge transport dynamics based on kinetic Monte Carlo to show charge transport regulation by dual-interface effect. (a) Three detailed examples of charge transport trajectories. (b) Heat map of 5000 charge transport trajectories. The electric field is 50 kV/mm directing from the oil region to the cellulose region.

Within the multiscale model, the hopping rates of all possible hopping processes in the interfacial morphology are calculated according to eq 8, and then, the charge transport dynamics are obtained by using the Kinetic Monte Carlo (KMC) method. The KMC simulations are performed using a single-charge approach. Figure 4a shows three detailed examples of individual KMC charge transport trajectories based on 100 hopping steps along the external electric field, where each polyline segment represents the hopping trajectory between two specific hopping sites.

Figure 4b presents a heat map of normalized count statistics of involved sites based on 5000 KMC trajectories. Injected into the oil region, the charges hop along the electric field with a spreading trend due to the diffusive, random walk nature of the hopping-type charge transport. The charges easily hop across interface 1 into the middle disordered hemicellulose region but are effectively trapped there: escaping back across interface 1 to the oil region or forward across interface 2 into the crystalline cellulose is blocked. Trapped charges transfer between sites within the middle region between two interfaces, leading to a deeper trajectory color in the heat map. It should be noted that the single-charge KMC simulation does not allow for conclusions regarding trap filling effects or other finite charge effects. As polymer dielectrics are usually operated in conditions of low carrier concentration, we expect such effects to be small and not to influence the fundamental mechanisms we discuss below.

**Figure 5:**
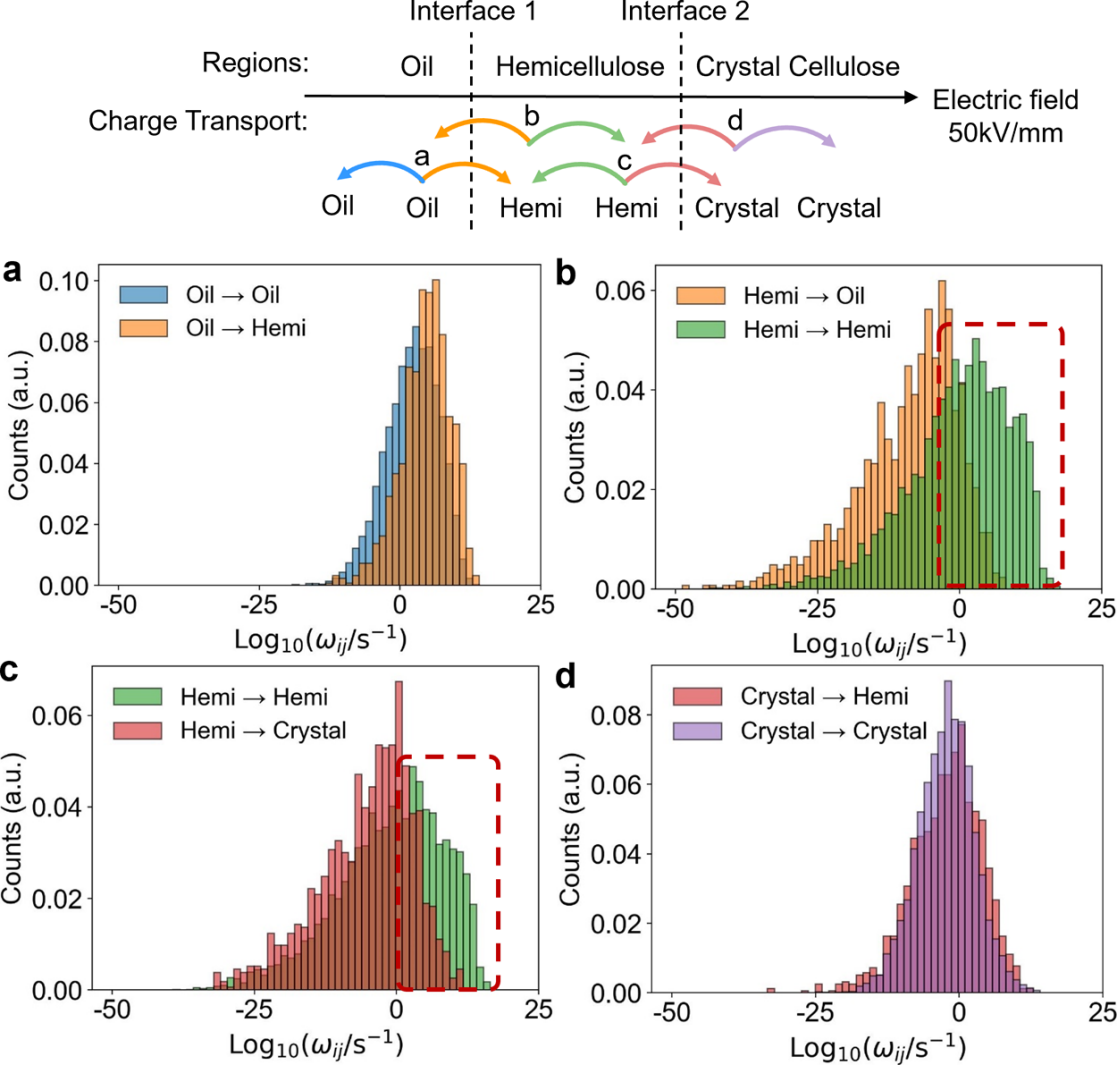
Statistical region-based results of charge hopping rate ω_*ij*_. (a,b) Results related to interface 1. (c,d) Results related to interface 2. The electric field is 50 kV/mm directing from the oil region to the cellulose region.

The charge trajectories from our multiscale approach explicitly reveal for the first time a dual-interface effect regulating charge trapping microscopically. This dual-interface effect can also be seen in more detail by an inspection of the interface-related characteristics of the charge hopping rates ω_*ij*_. In Figure 5, we show rate distributions classified according to the involved regions: oil, hemicellulose, and crystalline cellulose, respectively, including directionality across interfaces, as indicated in the schematic diagram at the top of the figure. First, Figure 5a shows the hopping rates when the charge is in front of interface 1, and we focus primarily on the high-rate subset of the distributions. The hopping rates of the Oil→Hemi processes are overall similar to the Oil→Oil related ones, with a few slightly higher values. This indicates that the charge transport inside the oil and from the oil into the hemicellulose is comparable and that there is effectively no local barrier for crossing the interface in the direction of the electric field. When the charge is in the hemicellulose region between two interfaces, the hopping rates of Hemi→Hemi are several orders of magnitude larger than Hemi→Oil, as shown in the red dotted box in Figure 5b. Therefore, after the initial injection of the charge into the hemicellulose, transport within this region is preferred, and the transfer back to the oil region is unlikely.

Focusing now on the processes involving interface 2, one can identify the opposite effect compared to the situation at interface 1. As one can see from the high-rate data in Figure 5c, hopping rates for crossing from the hemicellulose into the crystalline region are significantly smaller than for transfer processes within hemicellulose. Consequently, not only is the back-transfer of the charges from the hemicellulose to the oil region across interface 1 suppressed, but also the forward-transfer along the direction of the electric field across interface 2.

In summary, the result of the interplay of both interfaces is an effective trapping of the charges in the hemicellulose region.

More than a simple blocking of charge transport by a single interface, this is a dual-interface charge transport regulation realized by the synergy of the two interface effects. This regulation of charge trapping is crucial for dielectric materials and aligns with the long-standing goals of dielectric design. Compared to the trapping site introduced by previous molecular unit or group modification methods, this tailored region between two interfaces can provide more charge trapping sites, thus realizing a more effective charge transport regulation. Practically, this dual-interface effect matches the two mainstream interface implementation methods and can be correspondingly constructed by surface modification of nanoparticles^12,19−23^ and tailoring multilayer structures.^12,24−28^ Representing two generalized interface types presenting broad application potential, the revealed two interface effects are worth further studies of the underlying mechanisms. The following in-depth exploration of these two interface effects is based on two key aspects of charge hopping rate: charge attracting ability measured by the ionization energy of individual molecules and the electronic coupling between two molecules.

### Characterization of Charge Attracting Ability and Driving Force for Charge Hopping

**Figure 6:**
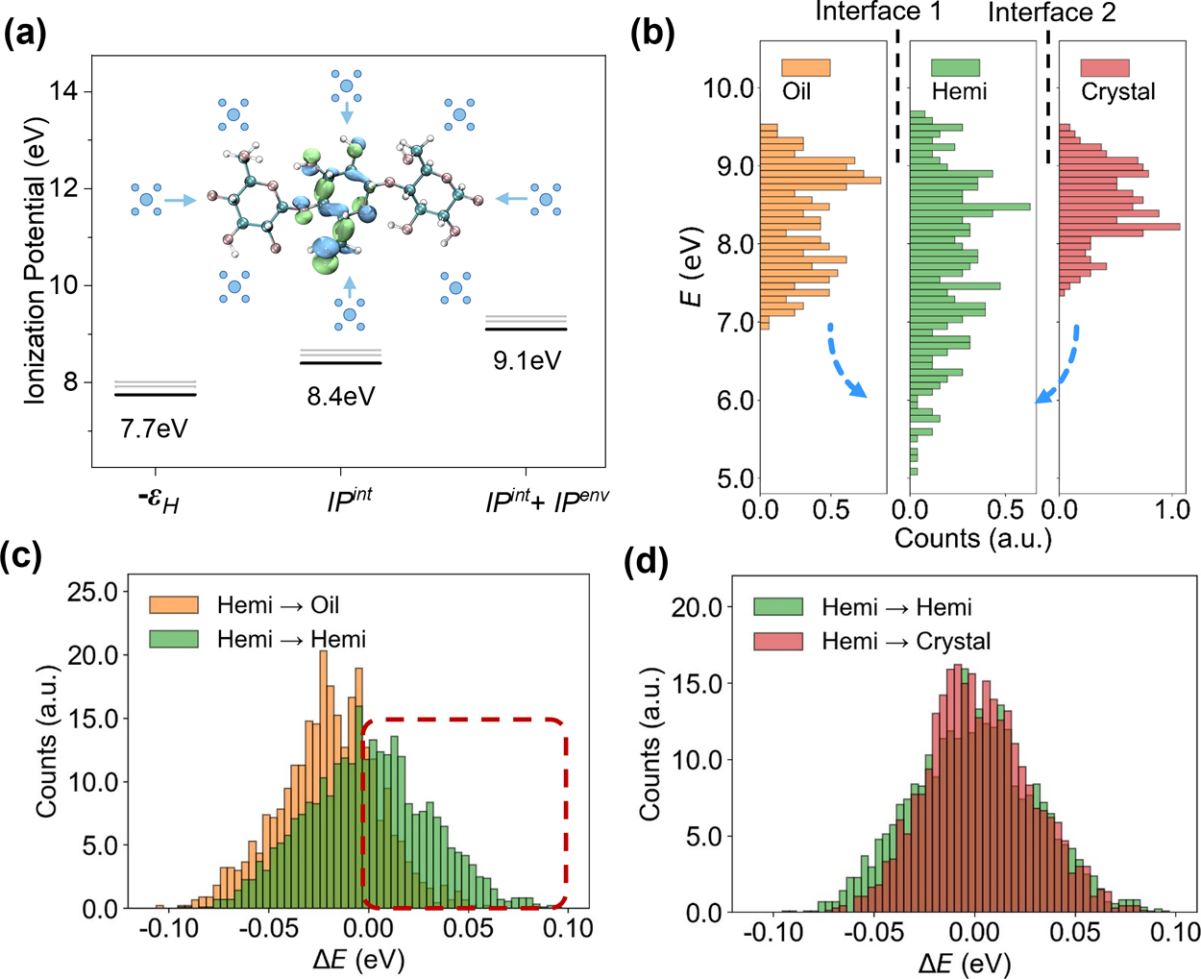
Characterization of charge attracting ability by site energy and interfacial analysis. (a) Schematic of the QM/MM calculation process of site energy. (b) Statistics of site energy *E* of different regions. (c) Region-based statistics of site energy difference Δ*E* related to interface 1. (d) Region-based statistics of site energy difference Δ*E* related to interface 2. No external electric field contribution is involved in the results in (a)–(d).

The charge attracting ability or ionization energy of a segment/molecule is often discussed in terms of frontier orbital energies obtained from effective single-particle quantum chemistry methods such as DFT.^6,13,34,36−38,44,45^ However, it is well-known that even for single molecules, these estimates are quantitatively and sometimes also qualitatively inaccurate. Single-molecule calculations also neglect the modification of the ionization energies due to the presence of other molecules in a morphology such as the oil-cellulose interface. Frontier orbital energies are also by definition proxies for vertical ionization energies, while the Marcus rate as in eq 8 requires the difference of adiabatic energies in Δ*E*_*ij*_. Instead, we here evaluate taking hole transport as an example, the ionization potential, i.e., the energy required to remove an electron from a neutral molecule^40,42^ from the difference of total energies of the molecules in charged (*E*_charged_) and neutral (*E*_neutral_) states:
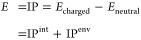
9In eq 9, we have split the ionization potential into internal (single molecule) and environment contributions. The schematic is shown in Figure 6a. In the previous reported studies,^6,13,34,36−38,44,45^ the charge attracting ability is characterized only by the original frontier orbital energy calculated by DFT, in which cases – ε_HOMO_ is applied as IP^int^. Here, we first get a proper IP^env^ from the calculations of DFT total energies in both states. Then, multiple environmental factors are accounted for in IP^env^ by a classical, atomistic model including electrostatic interactions and polarization effects. Taken together, the site energy is therefore calculated in a quantum-classical (quantum mechanics/molecular mechanics, QM/MM) setup whose details can be found in the “Methods” section.

The distribution of site energies, or the density-of-states (DOS) for charge transport, calculated according to eq 9 , is shown split into the three regions of the oil-cellulose interface in Figure 6b. To show the properties of the material itself, the results do not include an externally applied electric field, which will be studied later. The hole charge carriers energetically favor relaxation to low values of IP. In general, the region containing the lowest site energy is in the hemicellulose region, and from purely energetic considerations on the respective DOS, this seems to indicate that charge carriers can easily hop into but are difficult to escape from the hemicellulose region. This suggests that the trapping of charge carriers in the hemicellulose region is purely energy-regulated across both interfaces. It should be noted that due to the complexity of the structure and the macroscopic nature of existing experimental methods like thermal simulated depolarization current, to our knowledge, presently, no experimental measurements at the required resolution that can be directly compared to our results. However, the advancement of scanning probe microscopy techniques indicates a promising direction for future experimental confirmation.^13^

However, inspecting the overall DOS is misleading in the context of hopping transport as the fundamental electron transfer process is short-ranged and driven by the energy difference among hopping pairs. It is therefore more instructive to consider the distribution of site energy differences, or driving forces, Δ*E*_*ij*_ as it enters the Marcus rate in eq 8.

Figure 6c,d shows the site energy difference Δ*E*_*ij*_ related to interfaces 1 and interface 2, corresponding to Figure 5b,c, respectively. Figure 6c shows that in the relevant part of the positive driving force, the site energy difference between Hemi and Hemi is indeed larger than the one between Hemi and Oil, as shown in the red dotted box. The stronger driving force to remain in the hemicellulose region than to transfer back to oil supports, with the conclusions drawn from the overall charge hopping rates shown in Figure 5b for interface 1, and it confirms that the trapping of charges on this interface is indeed energy-regulated.

However, at interface 2 between hemicellulose and crystalline cellulose, the respective driving forces distributions of the related charge transport, as shown in Figure 6d, are similar. This lack of an effective energetic barrier is surprising given the pronounced differences in the respective rates in Figure 5c and excludes energy-regulation as a mechanism for trapping the charge carriers in the hemicellulose region at interface 2. In summary, the results in Figure 6c,d indicate that the site energy difference significantly contributes to the interface effect of interface 1 but not much to interface 2. To explain the interface effect at interface 2, we will consider the often-overlooked electronic coupling in the following.

**Figure 7:**
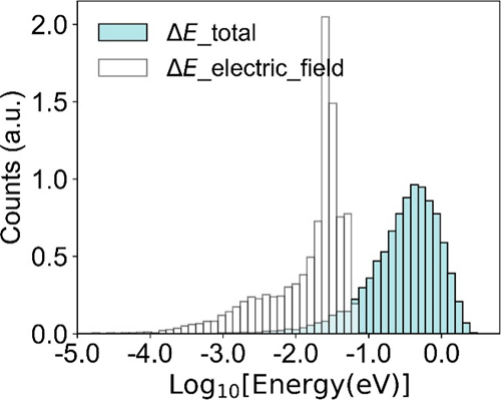
Comparison of the electric field component of Δ*E* and the entire Δ*E*.

Before turning to this analysis, in the context of driving forces, we briefly discuss the model of a shielding effect by charge accumulation, which is currently a popular explanation for the interface effect. When charges get trapped (or at least slowed down) at the interface, their presence creates an additional electric field that affects the other dynamic carriers. Instead of simulating these effects explicitly, we vary the interface-associated electric field during site energy difference calculations (the related principle can be found in the “Methods” section). Figure 7 shows a comparison between the total driving force in the whole system for an external field of 50 kV/mm and the contribution of the external field, demonstrating that the electric field is not the dominant factor in the site energy difference.

**Figure 8:**
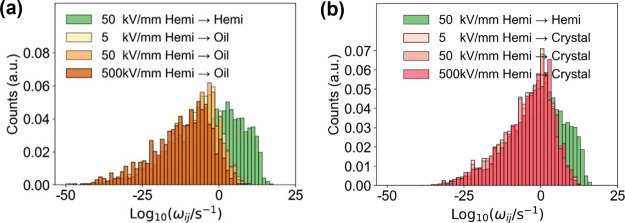
Influence of electric field on charge hopping rate related to the interface. (a) Influence of electric field on the hopping rate related to interface 1. (b) Influence of electric field on hopping rate related to interface 2.

In Figure 8a,b, we focus on interface 1 and interface 2, respectively, and strengthen (500 kV/mm) or weaken (5 kV/mm) the electric field during the calculation of interface crossing rates. With the increase in the electric field, the rate distributions shift toward lower values when the hopping is backcrossing in Figure 8a, and shift toward higher values when the hopping is forward-crossing in Figure 8b. It should be noted that the electric field effect is significant itself, as the rate of shifting is shown in the logarithmic coordinates. However, the relative distribution differences between hemi–hemi and hemi-oil or between hemi–hemi and hemi-crystal are not dramatically affected by the electric field. This implies that the shielding effect is insufficient to explain the interface effect, at least the observed trapping behavior in our interface system, for which the energetic factor discussed above could be more important.

### Crucial Role of Molecular Electronic Coupling for Interface Effect Hopping

Beyond the driving force of site energy difference Δ*E*_*ij*_, electronic coupling *J*_*ij*_ in eq 8 is the second key quantity influencing charge hopping rates. It measures the quantum-mechanical interactions of the localized electronic states identified with the frontier orbitals of two hopping sites, acting as a ‘bridge’ for charge hopping, as the HOMO isosurface of the dimer shown in Figure 3b②. Despite its essential position, electronic coupling is often overlooked in the previous related studies, where a comprehensive theoretical framework for charge transport is lacking. The essence of the interface effect on charge transport is the interplay between the nanoscale electronic process and the material morphology structure, therefore, the electronic coupling is the key to deconstructing this multiscale interplay.

**Figure 9:**
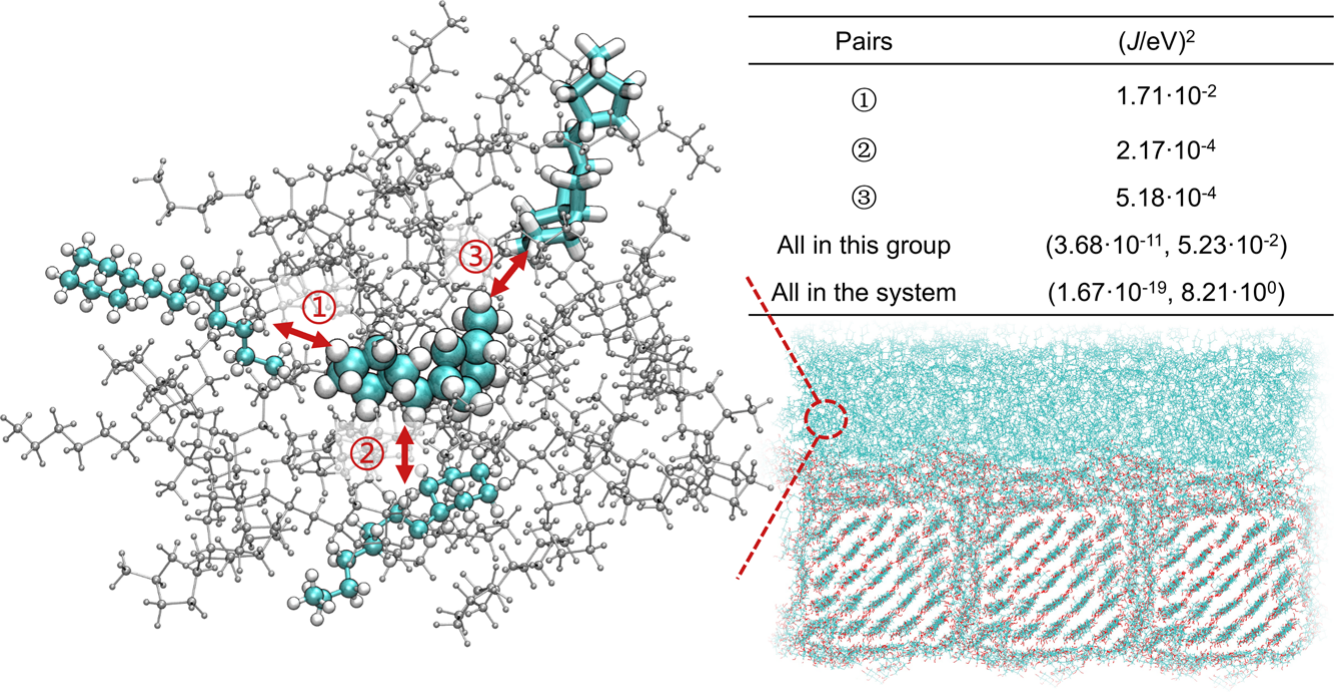
Sensitivity of electronic coupling. In the example group on the left side, the molecule pairs ① and ② consist of the same types of molecules but with different relative orientations, pair ① and ③ consist of different types of molecules. The right side is the summary of electronic coupling values.

Using an efficient method, we calculate the electronic coupling for all possible charge transport site pairs in the morphology. The basic calculation principle is described in the “Methods” section. Electronic coupling is highly sensitive to molecule types, relative orientation, and distance.^74,75^Figure 9 highlights this sensitivity with a specific group of pairs, where the centered highlighted molecule is the fixed component of all pairs, while the surrounding two layers of molecules with blue or gray color are the second components of pairs. Despite visually similar distances between the components of pairs ①, ②, and ③, differences in relative orientation and molecule types result in electronic coupling values varying by two orders of magnitude. Across the interface morphology, results span 19 orders of magnitude, indicating the electronic coupling’s significant influence on charge transport and its expected critical role in interface effects.

**Figure 10:**
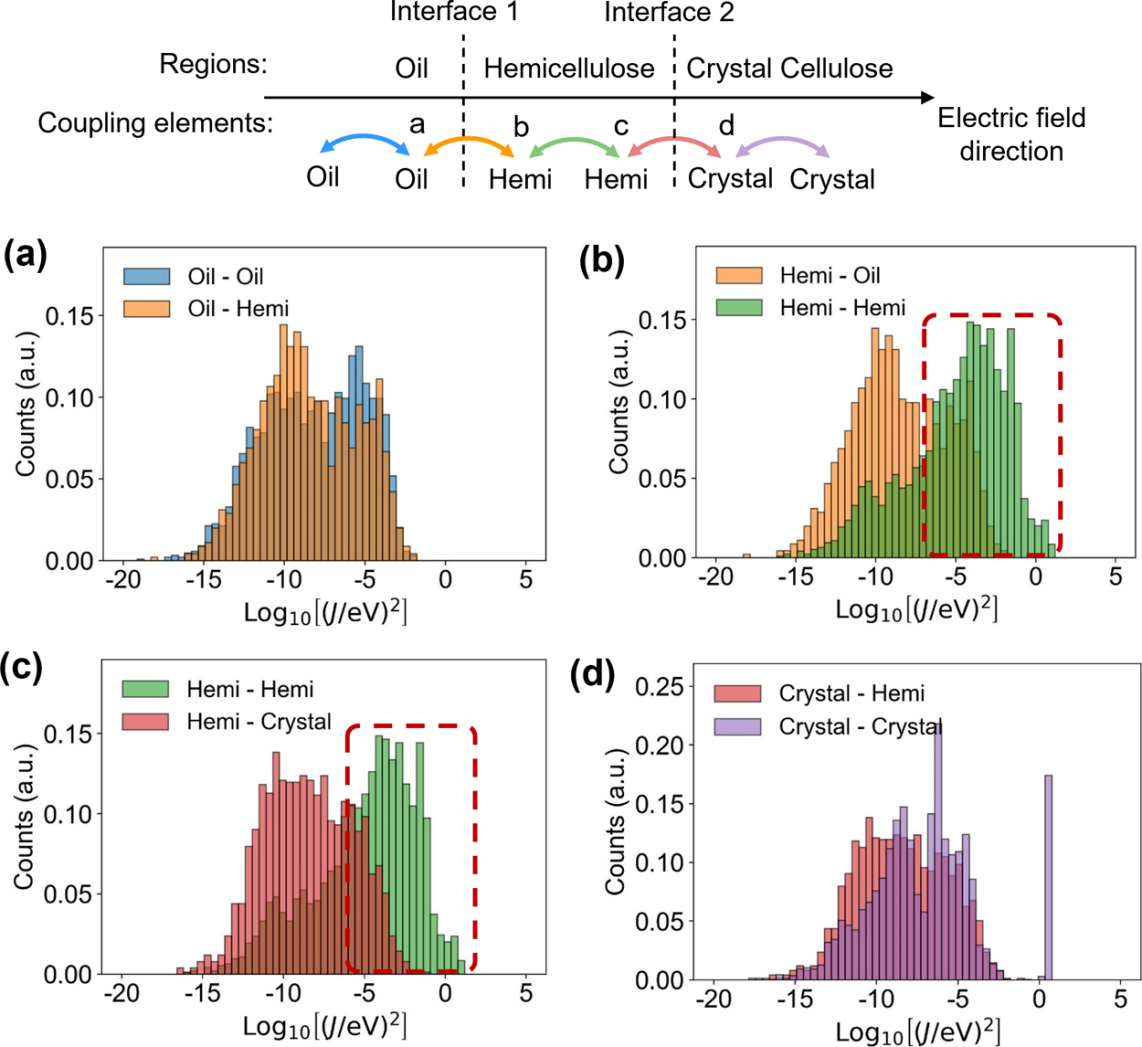
Region-based statistical results of electronic coupling *J*. (a,b) Results related to interface 1. (c,d) Results related to interface 2.

Figure 10 presents the distribution results of electronic coupling *J*. Figure 10a,b is related to charge hopping across interface 1, while Figure 10c,d is related to interface 2. The region-based results align with charge hopping rate statistics in Figure 5. Figure 10b shows that the electronic coupling for hopping across interface 1 is significantly lower than the one for hopping within the middle disordered hemicellulose region between two interfaces. This difference, combined with the site-energy differences discussed above, leads to a hindrance of charge back-transfer across interface 1, and favors transfer inside the disordered region. Figure 10c shows that the electronic coupling for hopping across interface 2 is also greatly lower than the one for hopping within the middle region. Here, however, this low coupling is crucial to explain the significantly lower rates for charge transfer into the cellulose crystal and the effective trapping of charges at the interface. Considering the weak contribution of site energy differences to the interface effect of interface 2 shown in Figure 6d, it is clear that charge trapping at interface 2 is coupling-regulated.

This notion of coupling-regulation vs energy-regulation is further corroborated by analyzing the different factors of the Marcus hopping rate. As shown in eqs 10 and 11, the Marcus rate is divided into two parts of the exponential term *G*_*ij*_(Δ*E*_*ij*_) and the remaining factor *F*_*ij*_(*J*_*ij*_), representing contributions of Δ*E*_*ij*_ and *J*_*ij*_ to ω_*ij*_, respectively:
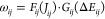
10with
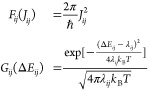
11

The statistical region-based results and the correlation analysis to the hopping rates shown in Figures S1–S4 of the Supporting Information.

The general distribution characteristics of the Δ*E*_*ij*_ contribution *G*_*ij*_(Δ*E*_*ij*_) are similar to the results of single Δ*E*_*ij*_, while the correlation between this contribution and the hopping rates is strong. On the other hand, the general distribution characteristics of the coupling factor *F*_*ij*_(*J*_*ij*_) are similar to the results of single *J*_*ij*_, while there is no obvious correlation between this contribution and the hopping rates. By comparing ω_*ij*_ in Figure 5 and the individual contributions in Figures S2 and S4, it is worth noting that the value range of *G*_*ij*_(Δ*E*_*ij*_) is comparable to ω_*ij*_’s, while the value range of *F*_*ij*_(*J*_*ij*_) is much narrower.

The correlation analysis between these two contributions and ω_*ij*_ is shown in Figures S3 and S5. There is a strong correlation between *G*_*ij*_(Δ*E*_*ij*_) and ω_*ij*_, while there is no obvious correlation between *F*_*ij*_(*J*_*ij*_) and ω_*ij*_. Based on the analysis above, it can be found that Δ*E*_*ij*_ has a determining effect on the specific distribution characteristics and basic position of ω_*ij*_, besides which *J*_*ij*_ plays a role in changing the mean order of magnitude of the hopping rate, intuitively shifting the position of ω_*ij*_ of each individual region as a whole. For example, in Figure 5c, the interface effect of interface 2 is directly reflected by the overall difference in relative position between the hopping rate from Hemi to Hemi (with green color) and the hopping rate from Hemi to Crystal (with red color). This relative position difference of hopping rate is primarily attributed to the shifting effect of electronic coupling (Figure 10c) rather than the site energy difference (Figure 6d). Therefore, it can be concluded that the electronic coupling dominates the interface effect of interface 2 on charge transport, while it amplifies the interface effect of interface 1, which is dominated by the site energy difference.

### Insights into Charge Transport Regulation

The microscopic charge transport dynamics, hopping rates, and analysis above provide insights into the interface effects on charge trapping regulation that contribute to the improvement of the dielectric strength of cellulose-oil composites. The corresponding mechanism is not merely a single interface effect that inhibits charge transport but rather a dual-interface effect arising from the synergy of two different interface mechanisms, resulting in a charge-trapping region between two interfaces. Different from traditional models that rely on individual trapping sites and are significantly influenced by trap filling or occupation effects, this regional trapping by a dual-interface synergy can achieve the regulation of more carrier transport. Moreover, while the thickness of the amorphous interphase is not the primary factor governing the observed mechanism, its intrinsic disorder could introduce additional trapping states, which may contribute to charge transport regulation in certain cases. Utilizing Marcus’ theory for localized-state polymer dielectrics, we find that the interface effects are governed by two crucial factors: charge-attracting ability (or ionization energy) and molecular electronic coupling. For the oil-hemicellulose interface (interface 1), both factors contribute to the interface effect, though energy regulation is the dominant mechanism. In contrast, for the hemicellulose-cellulose interface (interface 2), the energetic driving forces for transfer within hemicellulose and across the interface to the cellulose crystal are quite similar due to the comparable monosaccharide building blocks. Here, electronic coupling plays a pivotal role in determining the charge hopping differences between the two interface components, leading to a coupling-regulated interface effect. The interaction between these two interfaces ultimately facilitates charge trapping. The energy-regulated and coupling-regulated mechanisms of these two representative interface effects offer a general perspective for understanding different types of interfaces, such as those between chemically distinct components (e.g., nanodoping interfaces) and those within all-organic composite dielectrics.

A detailed analysis of site energy and electronic coupling offers theoretical support for understanding the mechanisms influencing dielectric strength. The role of charge-attracting ability has been associated with dielectric performance enhancement, as seen in studies introducing high-electron-affinity molecular semiconductors^33^ or electronegative molecular groups.^37^ Additionally, recognizing the role of molecular electronic coupling in hopping-type charge transport for polymer-based dielectrics provides further insights into this process. For instance, research on spiral-structured dielectric polymers has linked charge transport regulation to free volume,^34^ which may be relevant given that free volume can be associated with diminished electronic coupling due to its exponential decay with distance. Our study contributes to a broader charge transport framework by incorporating this concept and extending its applicability to interface effects in complex dielectric systems.

Understanding interface effects on charge transport provides a useful perspective for designing and optimizing advanced dielectrics. First, clarifying the synergy of interface mechanisms helps refine strategies for material modification. For example, enhancing cellulose-oil interfaces has been associated with an increase in the dielectric strength of cellulose dielectrics through simple impregnation. Similarly, introducing nanoscale interfaces via deposition layers has been shown to improve dielectric capacitive performance.^28^ Second, these multiscale insights support practical modification strategies by expanding available approaches. Recognizing the role of electronic coupling allows for more flexibility in modifying dielectric properties, which is particularly relevant for all-organic composites where charge-attracting ability alone may be insufficient due to the chemical similarity of components,^6,27,33^ as reflected in the mechanism of interface 2 in this work. Additionally, the dual-interface effect on charge trapping regulation may inform interface design strategies, where interacting interfaces can be implemented through surface modification of nanoparticles^15,19−23^ or by tailoring multilayer structures.^15,24,26−28,76^ Finally, from a methodological perspective, this work presents an approach to studying charge transport in complex molecular systems by integrating multiple theoretical and computational techniques. The combination of these methods enhances flexibility in analysis, making multiscale charge transport simulations a useful tool for exploring dielectric materials with energy-related applications.

## Conclusions

This work presents a structured framework for investigating charge transport in complex molecular systems, offering insights into fundamental processes and emergent mechanisms that may inform material development. By integrating molecular dynamics, quantum chemistry, kinetic Monte Carlo, and Marcus theory, we adopt a multiscale approach to examine hopping-type charge transport in polymer dielectrics. Using this approach, our study identifies interfacial charge trapping arising from two synergistic interface effects and explores their underlying mechanisms. The insights gained contribute to a broader understanding of interface effects, supporting more flexible strategies for interface engineering and material optimization. More broadly, this work provides a perspective for discussing charge transport in polymeric materials within a hopping transport framework, shifting focus from bandgap-centric interpretations toward a more proper theoretical foundation. The findings demonstrated here offer a basis for further studies and may assist in the development of polymer materials with tailored electrical properties.

## Supporting Information Available

Results of contribution of Δ*E*_*ij*_ and *J*_*ij*_ to ω_*ij*_ and the correlation analysis
