# Supplementary material for: Unveiling Synergistic Interface Effects on Charge Trapping Regulation in Polymer Composite Dielectrics through Multiscale Modeling
Source: J Phys Chem B. 2025 Apr 23;129(17):4216–28. doi: 10.1021/acs.jpcb.4c08661 (PMC12051192; doi:10.1021/acs.jpcb.4c08661)
Supplement: Supplementary file 1 — jp4c08661_si_001.pdf [file jp4c08661_si_001.pdf]

Supporting Information

Unveiling Synergistic Interface Effects on Charge Trapping Regulation in Polymer Composite Dielectrics through Multiscale Modeling

Haoxiang Zhao,[†,‡,¶] Lixuan An,[§] Daning Zhang,[†] Xiong Yang,[†] Huanmin Yao,[†] Guanjun Zhang,[†] Haibao Mu,[*,†] and Björn Baumeier[*,‡,¶]

†*State Key Laboratory of Electrical Insulation and Power Equipment, School of Electrical Engineering, Xi'an Jiaotong University, Xi'an 710049, China*

‡*Department of Mathematics and Computer Science, Eindhoven University of Technology, P.O. Box 513, 5600MB Eindhoven, The Netherlands*

¶*Institute for Complex Molecular Systems, Eindhoven University of Technology, P.O. Box 513, 5600MB Eindhoven, The Netherlands*

§*KERMIT, Department of Data Analysis and Mathematical Modelling, Ghent University, Ghent 9000, Belgium*

E-mail: haibaomu@mail.xjtu.edu.cn; b.baumeier@tue.nl

Results of contribution of ΔE_{ij} and J_{ij} to ω_{ij}, and the correlations

Based on the description in the main text, the Marcus rate

$$\omega_{ij} = \frac{2\pi}{\hbar} \frac{|J_{ij}|^2}{\sqrt{4\pi\lambda_{ij} k_\mathrm{B} T}} \exp\left[-\frac{(\Delta E_{ij} - \lambda_{ij})^2}{4\lambda_{ij} k_\mathrm{B} T}\right] \tag{S1}$$

is split into two parts: the exponential term

$$G_{ij}(\Delta E_{ij}) = \frac{\exp\left[-\frac{(\Delta E_{ij} - \lambda_{ij})^2}{4\lambda_{ij} k_\mathrm{B} T}\right]}{\sqrt{4\pi\lambda_{ij} k_\mathrm{B} T}} \tag{S2}$$

and the remaining factor

$$F_{ij}(J_{ij}) = \frac{2\pi}{\hbar} J_{ij}^2. \tag{S3}$$

The factors $G_{ij}(\Delta E_{ij})$ and $F_{ij}(J_{ij})$ represent the contributions of site energy difference and electronic coupling to ω_{ij}, respectively. The statistical region-based results of $G_{ij}(\Delta E_{ij})$ and $F_{ij}(J_{ij})$, and the correlation analysis to the hopping rates shown in Figs. S1 to S4.

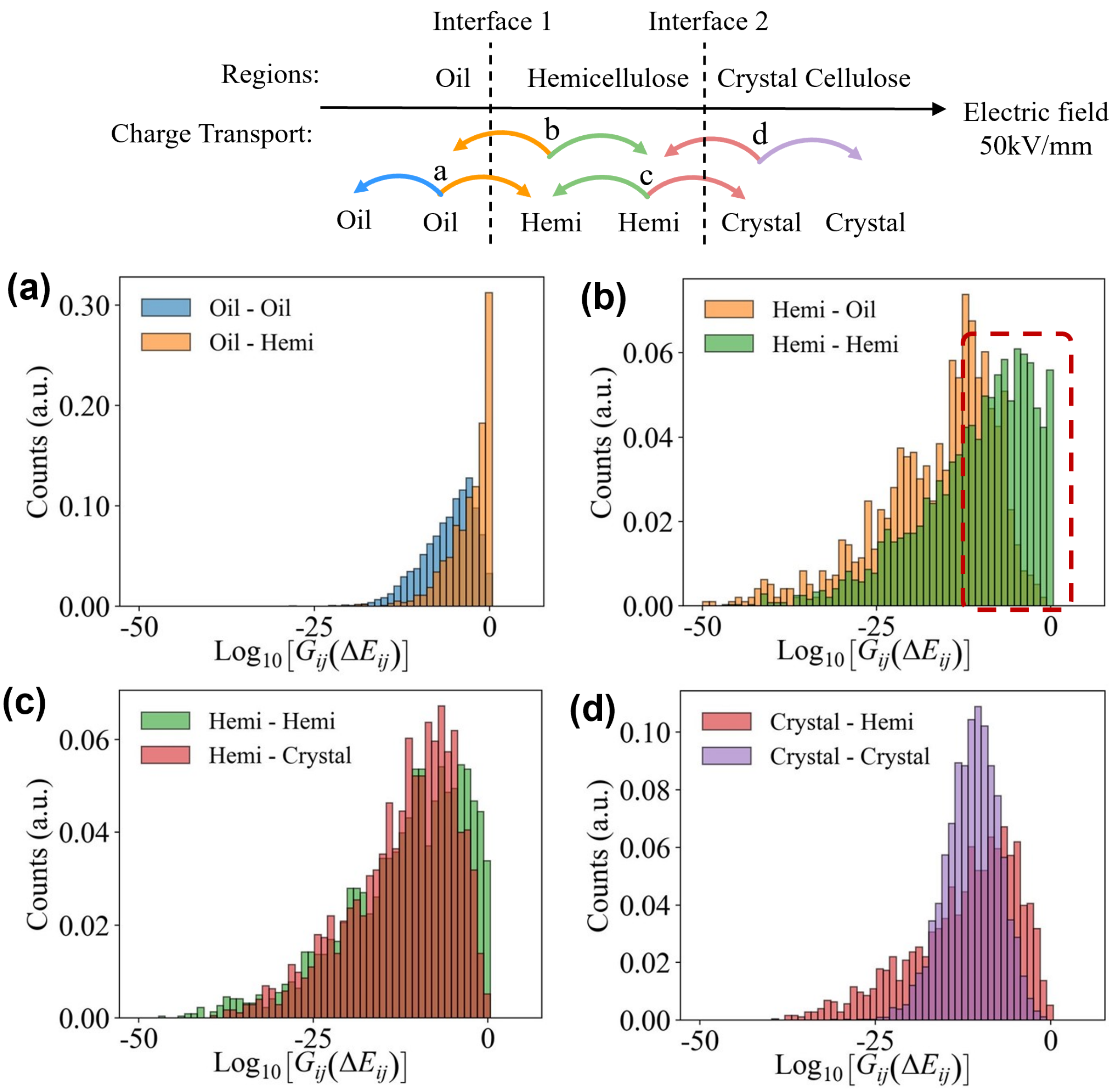

Figure S1: Statistical region-based results of $G_{ij}(\Delta E_{ij})$, the ΔE contribution to the hopping rate ω_{ij}.

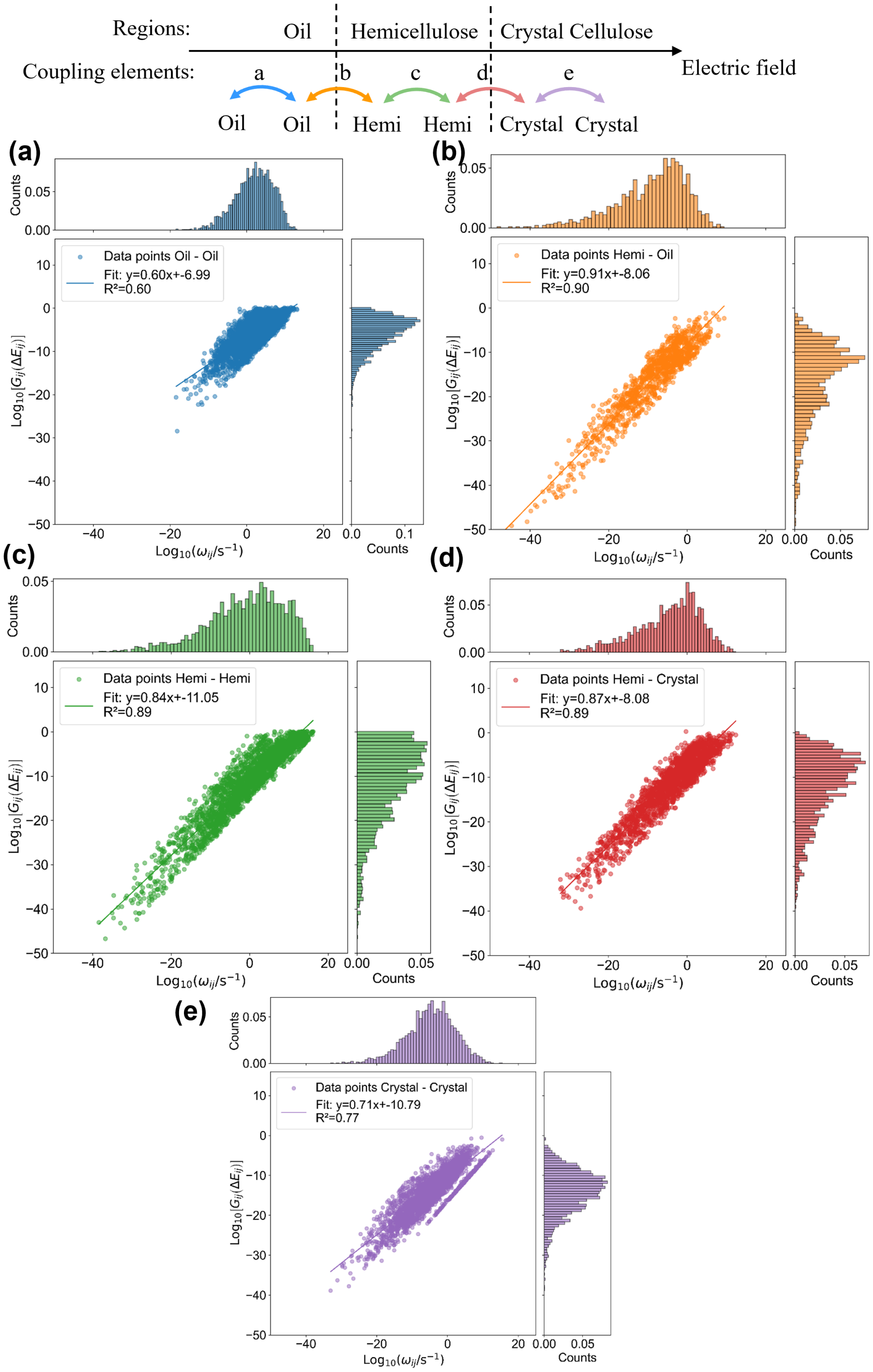

Figure S2: Correlation between the ΔE contribution $G_{ij}(\Delta E_{ij})$ and the hopping rate ω_{ij}.

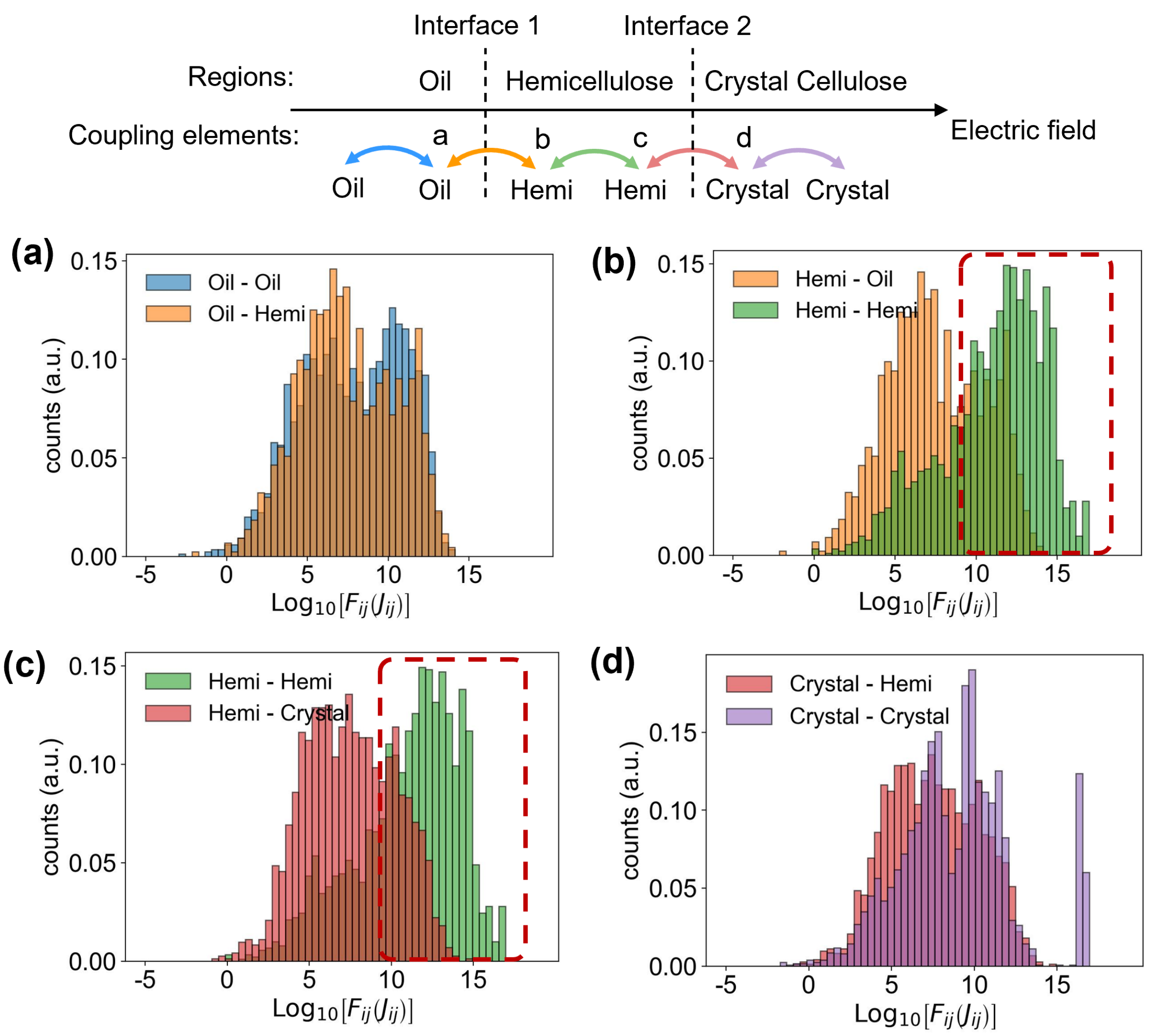

Figure S3: Statistical region-based results of $F_{ij}(J_{ij})$, the J contribution to the hopping rate ω_{ij}.

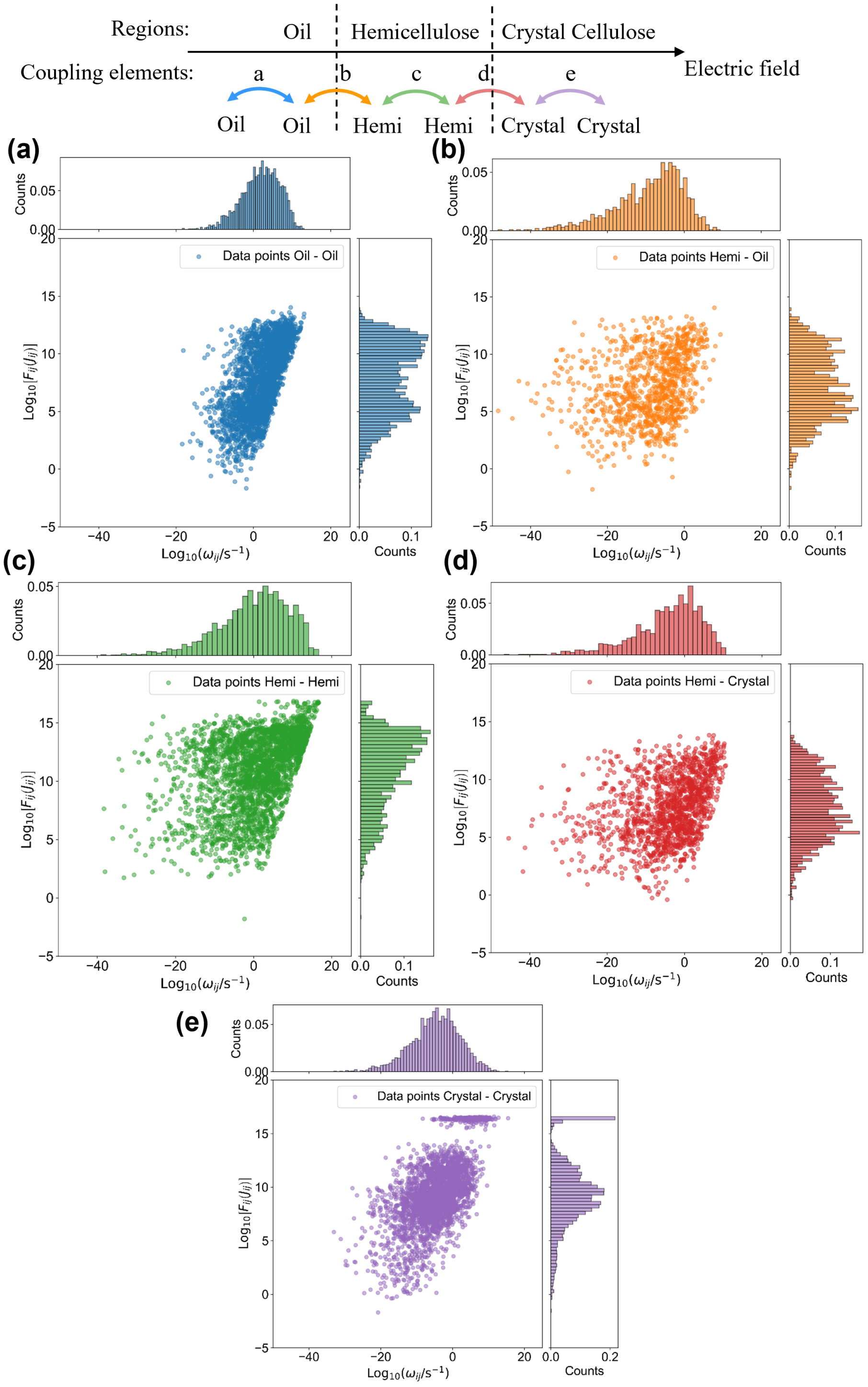

Figure S4: Correlation between the J_{ij} contribution $F_{ij}(J_{ij})$ and the hopping rate ω_{ij}.
